# Decreasing Rates of Fracture-Related Hospitalization With Primary Biliary Cholangitis: Insights From the Nationwide Inpatient Sample

**DOI:** 10.7759/cureus.25001

**Published:** 2022-05-14

**Authors:** Zaid Ansari, Ishani Shah, Abhishek Bhurwal, Harsh Mehta, Surabhi Uppal, Indu Srinivasan, Savio Reddymasu, Keng-Yu Chuang

**Affiliations:** 1 Internal Medicine, Creighton University School of Medicine, Phoenix, USA; 2 Internal Medicine, Creighton University School of Medicine, St. Joseph's Hospital and Medical Center, Phoenix, USA; 3 Gastroenterology, Robert Wood Johnson University Hospital, New Brunswick, USA; 4 Internal Medicine, Saint Barnabas Medical Center, New Jersey, USA; 5 Internal Medicine, Creighton University School of Medicine/St. Joseph's Hospital and Medical Center, Phoenix, USA; 6 Gastroenterology, Valleywise Health Medical Center, Phoenix, USA; 7 Gastroenterology, Creighton University School of Medicine, St. Joseph's Hospital and Medical Center, Phoenix, USA; 8 Internal Medicine/Gastroenterology, Valleywise Health Medical Center, Phoenix, USA; 9 Internal Medicine/Gastroenterology, Creighton University School of Medicine-Phoenix Program, Phoenix, USA

**Keywords:** bone metabolic disease, osteoporosis, hospitalization, fractures, primary biliary cholangitis

## Abstract

Introduction

Primary biliary cholangitis (PBC) is associated with an increased risk of developing fractures. Current guidelines recommend measures that can help prevent the development of fractures in these patients. The purpose of this study was to trend the rates of hospitalizations related to fractures and their burden on healthcare.

Methods

We performed a retrospective, cohort study of adults hospitalized in the United States with PBC between 2010 and 2014. Patients were identified using the Nationwide Inpatient Sample (NIS). Temporal analysis of PBC patients with a co-diagnosis of hip, vertebral, or wrist fractures (the study group) was performed with regards to the total number of inpatient admissions, inpatient mortality, length of stay, and total charges associated with hospitalization. Descriptive analyses were performed using the *t*-test for continuous data and the *chi-square* test for categorical data.

Results

During the five-year study period, there were 308,753 hospitalizations for PBC. There has been a downward trend (p=0.02) in fracture-related admissions among patients with PBC during this study period. Length of stay was higher in the PBC-fracture group (10.85 days vs 7.36 days; p<0.001). Total hospitalization charges were higher among the PBC-fracture patients when compared to the control group ($98,444 vs $72,964; p=0.004).

Conclusion

There has been a gradual reduction in the rate of fracture-related hospitalizations in patients with PBC. However, patients with PBC who have fractures have increased the utilization of health care resources as compared to their cohort admitted for reasons other than for a fracture.

## Introduction

Primary biliary cholangitis (PBC) is an immune‐mediated disease that involves small bile ducts of the liver and biliary tree. Apart from rare variants, its course is generally slow but tends to be progressive, and it can progress to cirrhosis, hepatocellular carcinoma, liver failure, and death. It is an important cause of liver-related morbidity, with the annual economic burden in the United States estimated to be between $69 and $115 million [1]. In addition, a recent population-based study estimates the mortality from PBC-related admission to be approximately 3% [2].

Fractures related to osteoporosis have a significant burden on healthcare resources with a retrospective study showing an average length of stay (LOS) of five days, ICU use of 7.4%, and a 1.5% mortality rate in a 60-day post-discharge day period [3]. A major consequence of the development of a fracture is the associated disability that makes the patient dependent on others resulting in excess death within one year of the fracture [4-5].

Metabolic bone disease is often seen among patients with chronic liver disease. It commonly manifests as osteoporosis and sometimes as osteomalacia. The pathogenesis of this phenomenon is complex and believed to be multifactorial: cholestasis can cause vitamin and calcium deficiencies, which in turn can cause secondary hyperparathyroidism and increased bone resorption; altered bilirubin metabolism can also cause osteoblast dysfunction, which contributes to decreased bone formation [6]. However, vitamin D supplementation to normal levels has not been shown to improve bone mineral density in PBC [7]. Keeping this in mind, the question arises - has there been any benefit from the widespread practice of calcium and vitamin D supplementation to prevent osteoporotic fractures?

Low bone density is a common occurrence among patients with PBC, with prevalence ranging from 20% to 50% [8-11]. In previous studies, age, weight, height, histologic stage of the disease, postmenopausal status, vitamin D deficiency, as well as severity and duration of liver damage have been identified as risk factors for osteoporosis among these patients [12-13]. In another population-based study, patients with PBC were found to have a two-fold fracture risk as compared to the general population, with an absolute excess fracture rate of 12.5 per 1000 person-years [14].

The aim of our study is to perform a national audit of fracture-related hospitalizations among PBC patients over a five-year period and to compare outcomes associated with mortality and healthcare utilization among these patients to PBC controls with non-fracture hospitalizations.

Part of this study has been presented at the following conference: The American Journal of Gastroenterology: October 2019 - Volume 114 - Issue - p S1592-S1593, doi: 10.14309/01.ajg.0000601200.73941.90.

## Materials and methods

Data source

The National Inpatient Sample (NIS) was created by the Agency for Healthcare Research and Quality (AHRQ) and is maintained by the Healthcare Cost and Utilization Project (HCUP) [15]. The NIS is the largest all-payer inpatient database available in the United States and constitutes all discharge data across the USA [15]. This data has been used to analyze national trends in outcomes for various diagnoses and major procedures, hospitalization rates, healthcare access, and disparity of care. The NIS represents approximately a 20 % stratified sample of all patients admitted to non-federal hospitals in the United States [15]. Each individual hospitalization is de-identified and maintained as a unique entry, with one primary discharge diagnosis and fewer than 24 secondary diagnoses. It also contains information on demographics, comorbidities, insurance status, primary and secondary procedures, hospitalization outcome, length of stay (LOS), and cost of care, with safeguards to protect patient, physician, and hospital privacy [15]. The NIS database has annual data quality assessments performed, thus guaranteeing the external and internal validity of the database [16].

Patient selection, study groups, and outcomes of interest

We used the NIS database to identify our study population during a five-year study period (2010-2014), using the International Classification of Diseases, Ninth Revision Clinical Modification (ICD-9-CM) diagnostic and procedure codes. All adults 18 years of age and above, hospitalized with a primary diagnosis of PBC (ICD-9-CM code 576.1) during the study period were included. From the PBC group, we identified patients with a secondary diagnosis of hip, wrist, or vertebral fractures, thereby creating two sub-groups: the study group, which consisted of PBC patients with fractures, and the control group, which consisted of PBC patients without fractures. The following comorbidities were included using appropriate ICD-9-CM codes: diabetes mellitus (250.0-250.9), hypertension (401.0, 401.1, 401.9), chronic kidney disease (CKD) (585.1-585.9), congestive heart failure (CHF) (428.0-428.9), inflammatory bowel disease (IBD), such as Crohn’s disease (555.0-555.2, 555.9) and ulcerative colitis (556.0-556.9), rheumatoid arthritis (RA) (714.0-714.9), osteoporosis (733.0), smoking status (305.1, V15.82), and pathological fractures (733.10-733.19). Our outcomes of interest were the length of hospital stay, inpatient mortality, and charges associated with hospitalization. Our study protocol was submitted to Valleywise Health IRB (Protocol No 2021-035), and it was determined to be exempt from IRB review.

Statistical analysis

Statistical analysis was performed using the STATA 13.0 SE software package (STATA Corp., College Station, TX). Continuous variables were analyzed using means and standard deviations. These were compared between the two groups using the t-test or Wilcoxon-sum test as considered appropriate. Categorical variables were described as frequencies (percentage) and analyzed using the two-way chi-square test. Statistical significance (p-value) was assigned at 0.05. Results were adjusted for age, gender, medical insurance, and comorbid conditions. Multivariate regression was performed to identify an independent association of fractures with inpatient mortality and length of stay among patients hospitalized with PBC.

## Results

**Figure 1:**
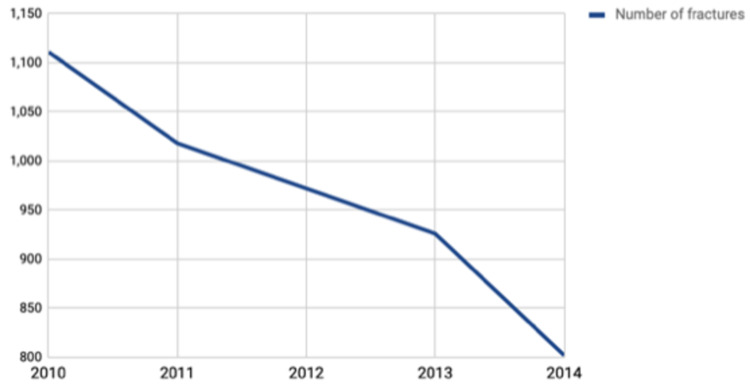
Trends of Fracture-Related Hospitalization Among Patients With Primary Biliary Cholangitis

During the five-year study period, a total of 308,753 patients with a discharge diagnosis of PBC were identified. Of these, 903 (0.3%) patients were found to have been hospitalized with a primary diagnosis of hip, vertebral or knee fractures and hence constituted our study group. The remaining 307,850 (99.69%) PBC patients hospitalized for primary reasons other than fractures constituted our control group. The trend of primary fracture-related hospitalizations over the five-year period from 2010 to 2014 manifested as a steady decline (p=0.02), as shown in Figure 1.

Patient characteristics and study outcomes

Table 1 outlines a comparison of demographic characteristics, associated conditions, comorbidities, and outcomes between the PBC-fracture and PBC-control groups. Compared to PBC-controls, patients in the PBC-fracture group were older (mean age 73.62 years vs 65.24 years; p=0.001), more likely to be females (51.92% vs 47.62%; p=0.24), and predominantly White (83.72% vs 69.62%; p=0.003). They were more likely to be insured by Medicare (75.14% vs 57.02%; p=0.001) and more likely to be admitted to hospitals in urban locations (93.03% vs 88.38%; p=0.02). Patients in both groups were comparable in terms of having long-standing comorbidities such as diabetes mellitus (23.03% vs 26.65%; p=0.26), hypertension 55.97% vs 54.6%; p=0.7), and CKD (14.03% vs 11.7%; P=0.03) as outlined in Table 1.

**Table 1:** Characteristics and Outcomes Compared Between Hospitalized PBC Patients With Fractures and PBC Controls Without Fractures PBC = Primary Biliary Cholangitis; IBD = Inflammatory Bowel Disease; ERCP = Endoscopic Retrograde Cholangiopancreatography

	PBC-FRACTURE	PBC-CONTROL	P value
MEAN AGE (years)	73.62 +/- 1.02	65.24 +/- 0.14	<0.001
SEX			0.24
Female (%)	51.92	47.62	
RACE			0.003
White (%)	83.72	69.62	
African American (%)	4.16	9.69	
Hispanic (%)	5.53	11.64	
INSURANCE			0.001
Medicare (%)	75.14	57.02	
Medicaid (%)	1.66	9.17	
Private insurance (%)	17.66	27.05	
ASSOCIATED CONDITIONS			
Smoking	12.19	20.87	0.004
Osteoporosis	15.73	4.09	<0.001
Pathological fractures	2.08	0.34	0.006
Rheumatoid arthritis	1.07	1.62	0.55
IBD	15.11	6.25	<0.001
Crohn’s disease	3.18	2.01	0.25
Ulcerative colitis	11.93	4.27	<0.001
PROCEDURES			
ERCP	6.75	9.82	0.16
MEAN LENGTH OF STAY (days)	10.85	7.36	<0.001
INPATIENT MORTALITY (%)	6.6	4.82	0.26
TOTAL HOSPITALIZATION CHARGES	$98,444	$72,964	0.004

Overall prevalence of IBD was higher in the PBC-fracture group (15.11% vs 6.25%; p<0.001), with ulcerative colitis (11.93% vs 4.27%; p<0.001) being more prevalent than Crohn’s disease (3.18% vs 2.01%; p=0.25). Although smoking (12.19% vs 20.87%; p=0.004) was less common among patients in the PBC-fracture group, both osteoporosis (15.73% vs 4.09%; p<0.001) and pathological fractures (2.08% vs 0.34%; p=0.006) were more prevalent among these patients.

Length of stay (10.85 days vs 7.36 days; p=0.001) was higher in the PBC-fracture group compared to the PBC-control group. Differences in inpatient all-cause mortality were higher in the PBC-fracture group but were not statistically significant (6.6% vs 4.82%; p=0.26). Total hospitalization charges in the PBC-fracture group were higher than those in the control group ($98,444 vs $72,964; p=0.004) (Table 1). On performing multivariate regression, it was revealed that fractures were independently associated with having increased odds of longer hospital stays (OR 0.4 [CI 0.23-0.55]; P<0.001) and higher mortality (OR 1.26 [CI1.20 - 2.61]); p=0.5), however, the relationship between fractures and mortality did not achieve statistical significance (Table 2).

**Table 2:** Multivariate Regression Analysis Determining the Independent Association of Fractures With Inpatient Mortality and Length of Stay Among Hospitalized PBC Patients PBC = Primary Biliary Cholangitis

	INPATIENT MORTALITY		LENGTH OF STAY	
	Odds Ratio (95% CI)	p>t	Coefficient (95% CI)	p>t
Fractures	1.26 (1.20 - 2.61)	0.5	0.4 (0.23-0.55)	<0.001

## Discussion

We used the nationwide inpatient sample (NIS) database for our study to demonstrate that 1) There has been a significant reduction in fracture-related admissions in patients with PBC during the study period; 2) Older age, white race, and female sex were risk factors for fracture-related hospitalizations; 3) A coexisting diagnosis of osteoporosis was more likely in patients with fracture-related hospitalizations; 4) There was increased utilization of healthcare resources in patients with fracture associated admissions.

The pathogenesis of osteoporosis in PBC is multifactorial, with osteoblast dysfunction and deficient bone formation being central to the process [13]. Studies have shown that serum levels of insulin-like growth factor-1 (IGF-1), an osteoblast growth factor, are lower in cirrhotics compared to controls [6,17]. Vitamin K has also been shown to stimulate osteoblastogenesis and inhibit osteoclastogenesis through carboxylation of osteocalcin [18]. Vitamin K levels may be decreased in severe cholestasis resulting in impaired osteoblast function. Osteoblast inhibition can occur by several mechanisms as well. Elevated levels of bilirubin, bile salts, lithocholic acid, and altered fibronectin production are thought to play a role in the inhibition of osteoblast function [19-21]. Increased bone resorption may also play a role in osteoporosis in PBC in certain populations such as post-menopausal women and men with hypogonadism [6,22-23].

In addition to all the mechanisms mentioned above, calcium and vitamin D deficiencies may develop in those with cholestatic liver disease leading to secondary hyperparathyroidism and increased bone resorption [6]. Studies have, however, found both decreased calcium, vitamin D, and parathyroid hormone (PTH) levels, as well as normal calcium, vitamin D, and PTH levels in PBC patients when compared to controls [21-22,24].

The association of osteoporosis with PBC has been well-established in the past with an estimated prevalence of 30% (between 20% and 50%) [8-11,25]. The incidence of fractures has been noted to be 14% over a two-year period and the prevalence has been reported between 10% and 20% [22,26-27]. Our study looks at the changing trends of fracture-related admissions during the study period (2010 to 2014) instead of focusing on the incidence or prevalence of fractures associated with PBC. These were found to have decreased significantly. Two mechanisms could be considered to explain our observations: 1. Over the study period, medical practitioners have increased screening efforts for osteoporosis in the general population, including those with PBC, and have improved managing patients with osteoporosis; 2. The screening and management of osteoporosis in PBC patients have improved. We, however, believe the first mechanism is less likely, as population-based studies have shown that there has been a decline in utilization of dual-energy X-ray absorptiometry (DEXA) scans since 2009 [28]. Bisphosphonate prescription also has decreased since the early 2000s [29]. Furthermore, statistics from the study period noted an increased incidence of total hip replacements for women aged over 45 [30]. This suggests overall worsening bone health for women during this time. The literature mentioned above does not support our hypothesis of better implementation of preventive measures. An additional limitation to this hypothesis is the limited awareness of PBC as an independent risk factor for osteoporosis among primary care providers (who are the front line in screening for treating osteoporosis ). Therefore, at this time, we cannot explain why we have observed this trend of decreased fracture-associated hospitalizations. Further data on osteoporosis prevention practices in the community are required to further investigate our hypothesis.

As in the general population, risk factors for osteoporosis in PBC have been known to include older age, female gender, early menopause, low BMI, and steroid use. We noted similar trends in our study. When compared with PBC-controls, PBC-fracture patients were older (mean age 73.62 years vs 65.24 years; p=0.001), more likely to be female (51.92% vs 47.62%; p=0.24), and predominantly White (83.72% vs 69.62%; p=0.003).

Furthermore, compared to PBC controls, patients with PBC fractures were more likely to have an associated diagnosis of osteoporosis (15.73% vs 4.09%; p=0.001). This suggests that osteoporosis plays a more significant role in fracture risk in PBC patients. Our analysis also supports the findings of similar studies that women with PBC are at higher risk for osteoporosis potentially based on the severity of cholestasis, advanced histological stage, and hepatic osteodystrophy as opposed to their menopausal status [22,31].

On reviewing the healthcare burden of fractures in PBC, we found that when compared to PBC-controls, the PBC-fracture group had a longer LOS (10.85 days vs 7.36 days; P=0.001), which resulted in a statistically significant increase in costs and health care utilization ($98,444 vs $72,964; p=0.004) but not statistical significance in difference for mortality. Marschall et al. found that patients with PBC had a significantly (p < 0.0001) increased risk of death from diagnoses related to digestive, cardiovascular, cancer, and respiratory causes [32]. Similarly, the major cause of death in PBC patients was noted to be digestive diseases related (22.9% vs. 2.5% in reference individuals) and most of them were liver-related (13.8% vs. 0.2%) as shown by Boonstra et al. as well [33]. Therefore, we are not surprised to see that fractures in patients with PBC do not have a substantial impact on mortality.

Interestingly, we also found that a coexisting diagnosis of inflammatory bowel disease (IBD) was noted to be seen more commonly in the PBC-fracture group vs the PBC-control group. While primary sclerosing cholangitis (PSC) is the most common hepatobiliary disorder associated with IBD, there have been reports of associations with PBC as well [34-35]. IBD has previously been independently associated with fracture-related hospitalizations [36]. The cause is multifactorial and includes chronic inflammation with cytokine-mediated bone resorption, glucocorticoid use, and vitamin D deficiency [23]. Our finding might be an indicator that the coexistence of both diseases has a higher likelihood of causing metabolic bone disease and eventual fractures. Our literature search did not find any studies looking at fracture risk in patients with both IBD and PBC, and it would be intriguing to see studies carried out to look into this hypothesis.

## Conclusions

In conclusion, there has been a steady decrease in the rate of fracture-related hospitalizations in patients with PBC. However, patients with PBC who suffer fractures utilize more health care resources as compared to those with PBC admitted for reasons other than a fracture. Increased implementation of measures to improve bone health in patients with PBC will hopefully continue to reduce the rate of fracture-related hospitalizations.
